# Intravenous ferric carboxymaltose versus oral ferrous sulphate for iron deficiency anaemia in pregnancy in Nigeria: a cost-utility analysis

**DOI:** 10.1016/S3050-5038(26)00049-X

**Published:** 2026-06

**Authors:** Opeyemi Rebecca Akinajo, Kristi Sidney Annerstedt, Maria Teresa Santos, Bosede Bukola Afolabi, Aduragbemi Banke-Thomas

**Affiliations:** a Department of Obstetrics and Gynaecology, Lagos University Teaching Hospital, Lagos, Nigeria; b Department of Global Public Health, Karolinska Institutet, Stockholm, Sweden; c Department of Public Health, Institute of Tropical Medicine, Antwerp, Belgium; d Department of Obstetrics and Gynaecology, Faculty of Clinical Sciences, College of Medicine, University of Lagos, Lagos, Nigeria; e Centre for Clinical Trials, Research, and Implementation Science, College of Medicine, University of Lagos, Lagos, Nigeria; f Maternal, Adolescent, Reproductive and Child Health Centre, London School of Hygiene & Tropical Medicine, London, UK

## Abstract

**Background:**

Iron deficiency anaemia (IDA), a prevalent condition in pregnancy, is often treated with oral iron, but with poor adherence. Evidence from trials has shown that ferric carboxymaltose, an intravenous iron therapy, is safe and highly effective. We aimed to conduct a cost-utility analysis of intravenous iron for moderate-to-severe IDA in pregnancy. This was a prespecified analysis of the Intravenous Versus Oral Iron for Iron Deficiency Anaemia in Pregnant Nigerian Women (IVON) trial.

**Methods:**

We developed a decision-tree model to evaluate the cost-utility of intravenous ferric carboxymaltose compared with oral ferrous sulphate for women with moderate-to-severe IDA during pregnancy in Nigeria. The model used data from the IVON trial, which was conducted in 11 health-care facilities in Nigeria and enrolled pregnant women aged 15–49 years between the gestational age of 20 weeks and 32 weeks with haemoglobin of less than 10 g/dL. By use of data from the IVON trial and secondary sources, we estimated intervention costs and did a base-case analysis to estimate the relative cost-utility of ferric carboxymaltose compared with ferrous sulphate. Model outputs included the total expected cost and health outcomes per pregnant woman for each intervention. Health outcomes were measured as disability-adjusted life-years (DALYs) averted. We calculated the incremental cost-utility ratio (ICUR) and compared it with the opportunity cost-based threshold. We considered ferric carboxymaltose as very cost-effective if the ICUR was less than 0·5 times the gross domestic product (GDP) per capita and cost-effective if it fell between 0·5 and 1·0 times the GDP per capita. The analysis focused on a time horizon of less than 1 year from a partial societal perspective. Additionally, we conducted sensitivity analyses to assess the robustness of findings in relation to parameter uncertainty. The IVON trial was registered with ISRCTN (ISRCTN63484804) and ClinicalTrials.gov (NCT 04976179) and is complete.

**Findings:**

In the base-case analysis, treatment with ferric carboxymaltose incurred a higher cost per pregnant woman (US$139·71) than treatment with ferrous sulphate ($104·33). Ferric carboxymaltose showed greater effectiveness by incurring fewer DALYs (0·0111) than ferrous sulphate (0·0377). The resulting ICUR was $1331·12 per DALY averted, which is between 0·5 and 1·0 times Nigeria's GDP per capita, making ferric carboxymaltose cost-effective. The ICUR was most sensitive to the acquisition costs of the ferric carboxymaltose vials, followed by the associated productivity costs. These costs exceeded the threshold when they surpassed $103·61 (for acquisition costs) and $49·75 (for productivity costs).

**Interpretation:**

Ferric carboxymaltose is a cost-effective intervention to reduce the maternal morbidity burden associated with IDA in pregnancy. Integration of ferric carboxymaltose into national guidelines for antenatal care and health insurance schemes, based on negotiated prices, will enhance health equity and improve outcomes for pregnant women.

**Funding:**

Bill & Melinda Gates Foundation.

## Introduction

Anaemia in pregnancy is a major public health issue, with a global prevalence of 36% and a particularly high prevalence in Africa of 43%.^1^ Nigeria, the most populous country in Africa, has one of the highest burdens of anaemia in pregnancy on the continent, at 45%.^1^ Iron deficiency anaemia (IDA) is the most common type of anaemia in pregnancy, with prevalence in Nigeria ranging from 25% to 45% across states.^2^ IDA increases the risks of poor pregnancy outcomes, including postpartum haemorrhage, postpartum anaemia, depression, and maternal mortality, and contributes to adverse neonatal outcomes such as preterm birth, low Apgar score, low birthweight, and perinatal or neonatal mortality.^3^ To minimise these risks, WHO recommends daily oral iron tablets as a standard preventive and therapeutic option to be prescribed during antenatal care.^4^ However, side-effects of oral iron, which include gastrointestinal symptoms such as nausea, vomiting, abdominal pain, diarrhoea, and constipation, limit adherence and lead to treatment failure.^5^, ^6^ Across Nigeria and other African countries, where adherence rates are broadly low, additional reasons relating to poor use of antenatal care, inability to pay for the tablets, missed doses, insufficient patient education, and inaccurate information have been attributed to non-adherence.^7^

**Research in context**

**Evidence before this study**

We searched the PubMed database to identify studies published between Jan 1, 2020, and Jan 31, 2025 that examined the cost-effectiveness and cost-utility of intravenous iron for treating anaemia in pregnancy. The search used the following terms: (cost OR cost-effectiveness OR cost-utility analysis) AND (anaemia in pregnancy OR maternal anaemia OR gestational anaemia OR iron deficiency anaemia in pregnancy) AND (intravenous iron OR parenteral iron OR iron infusion). The search did not have any language restrictions. Eligible studies were required to include a comparative analysis of both costs and effectiveness or a cost and utility analysis, and they must have reported results as an incremental cost-effectiveness ratio. Our search identified two full-text articles, one abstract, and one protocol for studies that evaluated the cost-effectiveness of intravenous iron, specifically intravenous iron sucrose, compared with oral iron therapy in pregnancy. Ray and colleagues used a cost-effectiveness analysis with a unidimensional outcome of safe delivery; this analysis does not allow a comprehensive understanding of the comparative benefit of the intervention, which is important for informing clinical and policy decisions. In another study by Saha and colleagues, a cost-utility analysis was done, reporting the cost per quality-adjusted life-year, which is rarely available in low-income and middle-income countries (LMICs). Disability-adjusted life-years (DALYs) are widely used, and are recommended for use in LMICs due to the availability of standardised disability weights from Global Burden of Disease studies and their use in facilitating cross-country comparisons. However, none of the studies identified in our search used DALYs as an outcome measure.

**Added value of this study**

The key added value of this study lies in its contribution to closing a substantial global evidence gap. Despite the increasing recognition of intravenous iron therapies for treating iron deficiency anaemia, no economic evaluation has yet assessed the cost-effectiveness of ferric carboxymaltose, an intravenous iron formulation that can be administered as a single dose with a better safety profile than other intravenous iron formulations previously investigated (eg, iron sucrose). This gap is even more crucial in Africa, where the burden of iron deficiency anaemia is among the highest globally and where such economic evidence is virtually non-existent. This study is the first to evaluate the cost-utility of ferric carboxymaltose compared with oral iron, not only in Nigeria or Africa, but globally. Our findings show that ferric carboxymaltose is more cost-effective than oral iron in Nigeria. These results provide much-needed, locally relevant evidence to inform health policy in Nigeria, with broader implications for other LMICs facing a similarly high burden of iron deficiency anaemia, where DALYS are more commonly used for decision making.

**Implications of all the available evidence**

The findings of this study build on existing research on economic evaluations in various health-care settings, showing that administering intravenous iron to pregnant women with iron deficiency anaemia is a cost-effective intervention. Specifically, we added to the existing literature by highlighting the cost-effectiveness of ferric carboxymaltose, an intravenous iron formulation that has been less studied in terms of economic evaluation compared with other intravenous iron formulations. However, to improve accessibility and affordability for this vulnerable population, costs associated with ferric carboxymaltose should be reduced. This finding is particularly important in sub-Saharan Africa, where countries such as Nigeria face substantial resource constraints, and a high prevalence of maternal mortality. The results emphasise the urgent need for context-specific strategies to make ferric carboxymaltose affordable and easily accessible to pregnant women, ultimately aiming to reduce the morbidity and mortality associated with iron deficiency anaemia in these regions.

An alternative therapy for IDA in pregnancy, which enables rapid iron replacement and is recommended as part of guidelines in high-income countries, is intravenous iron.^8^ To date, various formulations of intravenous iron, including iron dextran, iron sucrose, ferric derisomaltose, and ferric carboxymaltose, have been used to treat IDA.^9^ However, for various reasons, including its higher cost compared to oral iron, intravenous iron in all its forms has yet to become a mainstay in low-income and middle-income countries (LMICs).^10^

Of the various formulations of intravenous iron, there has been increasing interest in deploying ferric carboxymaltose in Africa.^11^ This interest is driven by the fact that ferric carboxymaltose can be given to pregnant women as a single dose and has been shown to have better efficacy and a better safety profile than other formulations.^12^, ^13^ Two recently concluded randomised controlled trials in Africa—one in Nigeria and the other in Malawi—showed that ferric carboxymaltose is safe and more effective in reducing the prevalence of IDA in pregnancy compared with the standard treatment of oral iron.^13^, ^14^ However, as with other intravenous iron formulations, the cost of ferric carboxymaltose can impose a substantial financial burden on pregnant women and their families, especially in settings where health care is commonly funded out of pocket and, for many, its cost can be prohibitive.^10^ Even in health systems where care is partly subsidised through health insurance schemes or public funding, its steep cost can be a barrier to its inclusion in essential health-care packages. Nonetheless, considering the evidence of the relative effectiveness of ferric carboxymaltose in reducing the risk of anaemia-related complications compared with oral iron,^13^, ^14^, ^15^ its comparative cost-effectiveness should be considered, especially in high-burden settings.

To date, only two economic evaluations have been published comparing intravenous iron with oral iron, both of which compare cost-effectiveness of iron sucrose formulation with oral iron in India.^16^, ^17^ However, one of the studies used a unidimensional natural unit,^16^ which does not allow comparison with other health economic evaluations,^18^ and the other used quality-adjusted life-years,^17^ which are not commonly used in LMICs. There remains a crucial knowledge gap in the cost-effectiveness of ferric carboxymaltose compared with oral iron globally and particularly in Africa, where no cost-effectiveness evidence of any sort has been published, despite the high burden of anaemia in pregnancy.^1^ Such evidence will be crucial for informing clinical decision making and efficient resource allocation. To address this gap, we conducted an economic evaluation alongside a clinical trial to estimate the incremental costs and comparative cost-utility of intravenous ferric carboxymaltose in comparison with oral ferrous sulphate in pregnant women with moderate-to-severe IDA in Nigeria.

## Methods

### Study design, participants, and procedures

Herein, we report the prespecified cost-utility analysis of the Intravenous Versus Oral Iron for Iron Deficiency Anaemia in Pregnant Nigerian Women (IVON) trial, an open-label type 1 hybrid effectiveness–implementation study carried out between Aug 10, 2021, and June 15, 2023.^19^ The primary outcomes of the IVON trial were maternal anaemia at 36 weeks' gestation and preterm birth at before 37 weeks' gestation, with analysis by intention to treat in participants with available data; these results are reported elsewhere.^19^ This study used a cost-utility analysis to assess the comparative costs and outcomes associated with intravenous ferric carboxymaltose (intervention) and oral ferrous sulphate (standard care) for the treatment of moderate-to-severe IDA in pregnancy. We reported our findings according to the CHEERS guidelines.^20^

The study was done in Nigeria, an LMIC with a gross domestic product (GDP) per capita of US$1596·64 as of 2023.^21^ The country has a population of over 200 million, of whom 30·7% were estimated to live below the poverty threshold of $2·15 per day, with out-of-pocket health expenditure accounting for 76·1% of health-care financing.^22^ The IVON trial was conducted in 11 health-care facilities in Nigeria's most populous and socioeconomically diverse states, Lagos and Kano, with Kano comprising a population with generally lower socioeconomic status and educational attainment than Lagos. The selected facilities spanned the three levels of care in the country (ie, primary, secondary, and tertiary care), providing a representative mix of health-care delivery settings (appendix 1 p 1).

Details of the selection of study participants and trial procedure have been published elsewhere.^19^ In summary, the IVON trial enrolled 1056 pregnant women aged 15–49 years between the gestational age of 20 weeks and 32 weeks with haemoglobin of less than 10 g/dL. Participants were randomly assigned in a 1:1 ratio to receive intravenous ferric carboxymaltose or oral ferrous sulphate (control).^13^ Participants in the ferric carboxymaltose group (n=527) were given a single infusion of intravenous ferric carboxymaltose, constituted as 20 mg/kg up to a maximum total dose of 1000 mg, diluted in 200 mL of 0·9% normal saline. The administration, which was done by skilled health-care personnel, took 15–20 min and the participants were closely monitored for 30 min afterwards. Participants in the ferrous sulphate group (n=529) received 200 mg tablets containing 65 mg elemental iron of ferrous sulphate three times daily from enrolment until 6 weeks after delivery, as per the standard practice for treating anaemia in pregnancy in Nigeria.^4^, ^13^

### Model design and parameters

**Figure 1:**
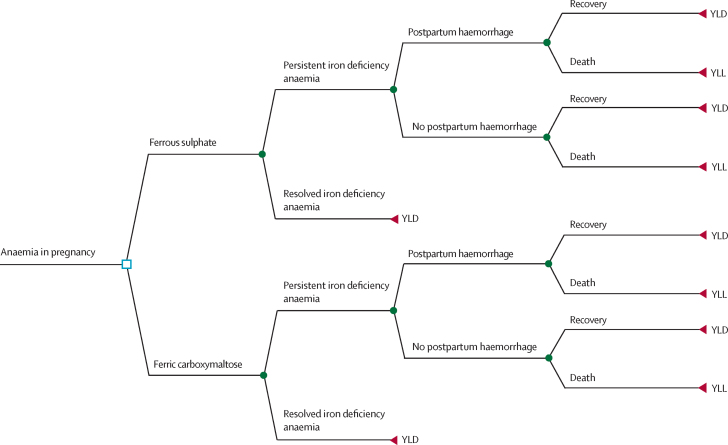
Decision tree for treatment with ferrous sulphate versus ferric carboxymaltose YLD=years lived with disability. YLL=years of life lost due to premature death.

A decision-analytic model with a cohort-based decision tree was developed by use of TreeAge Pro 2025 R1 (TreeAge Software, Williamstown, MA, USA) to conduct a cost-utility analysis comparing the treatment effect of ferric carboxymaltose with ferrous sulphate for treating IDA in pregnancy. The use of a decision-tree model is particularly suitable for delineating mutually exclusive clinical pathways in which IDA outcomes occurred as progressive events through the pregnancy-to-postpartum period without recurring transitions between clinical states.^18^ The structure of the model reflects the clinical pathway of pregnant women diagnosed with moderate-to-severe IDA and focuses exclusively on maternal outcomes, as the IVON trial did not show statistically significant differences in neonatal outcomes between the ferric carboxymaltose group and ferrous sulphate group. The decision tree begins with a pregnant woman receiving either ferrous sulphate or ferric carboxymaltose (figure 1). Each treatment branch leads to one of two initial outcomes from pregnancy until postpartum: resolution of IDA (indicating treatment success) or persistent IDA (indicating treatment failure). For women with persistent IDA, the model then captures the possibility of having postpartum haemorrhage and maternal death. To reflect the full health effect, we also considered the disability burden associated with treatment-related events, such as gastrointestinal symptoms from ferrous sulphate and administration reactions from ferric carboxymaltose, as reported in the literature.^3^, ^5^ These events were included to account for the clinical consequences and cost implications of unresolved anaemia during pregnancy.^16^, ^17^ All terminal nodes represent mutually exclusive health outcomes, to which costs and disability-adjusted life-years (DALYs) were assigned. We used DALYs as a composite measure because they provide a comprehensive understanding of the morbidity and mortality associated with anaemia in pregnancy. This measure is particularly appropriate for LMICs, as it enables feasible, comparable estimation of health outcomes with readily available data. Overall, the model structure was designed to reflect real-world care pathways in routine clinical practice, with potential outcomes ranging from recovery to death. The structure of the model was kept as simple as possible to reflect the clinical pathway of pregnant women diagnosed with moderate-to-severe IDA. Only key health states and outcomes relevant to the comparison were included. Additional complexities (eg, long-term complications) were not incorporated into the model as they were unlikely to substantially affect the results.

For the base-case scenario, we used context-specific evidence from the IVON trial, with the assumption that all pregnant women start antenatal care in their first trimester, having at least eight antenatal care visits and a 6-week postpartum period, in line with WHO recommendations.^4^ Additionally, each participant was assumed to have made two postnatal care visits, leading to ten scheduled visits resulting in 245 days of ferrous sulphate use for participants in the ferrous sulphate group or 1 day of ferric carboxymaltose for participants in the ferric carboxymaltose group. We considered an overall model time horizon of 46 weeks, reflecting the duration of both pregnancy (40 weeks) and postpartum periods (6 weeks).

### Cost parameters

We adopted a partial societal perspective to account for the costs that pregnant women and their caregivers incur when accessing care and the hospital costs associated with providing that care. We collected cost data (appendix 1 pp 3–5) from all participants, skilled health-care personnel, and health-care facility records during the antenatal and postpartum periods of the IVON trial. We used a microcosting approach based on the IDA pathway model described to account for direct (medical and non-medical) and indirect costs for pregnant women.^18^ Additionally, we used a time-based activity approach to estimate skilled health-care personnel time for each activity through direct observations. We used data from each facility's records (appendix 1 pp 3–5) to estimate cost of salaries by calculating hourly wage rates and multiplying them by the time spent on each activity. Individual salary was collected and the average was used for base-case analysis. The direct medical costs for the ferric carboxymaltose group included the cost of ferric carboxymaltose vials, administration costs (eg, consumables for administration, skilled health-care personnel's time before, during, and after administration, including extra time for observation of adverse events), costs of ferric carboxymaltose-related disabilities, facility-based costs (eg, costs of emergency readiness measures and capital costs of monitoring and infusion equipment), costs of postpartum haemorrhage, transport costs, and productivity costs (appendix 1 pp 3–5). The costs for the ferrous sulphate group comprised the cost of the ferrous sulphate drug course (eg, ferrous sulphate tablets and commonly used water in sachets for taking medicines), administration costs (eg, skilled health-care personnel's time for prescribing and dispensing ferrous sulphate throughout pregnancy and postpartum), costs of ferrous-sulphate-related disabilities, costs of postpartum haemorrhage, transport costs, and productivity costs (eg, time spent by skilled health-care personnel and pregnant women on unscheduled antenatal care visits, pharmacist dispensing, and laboratory analysis). Cost data for most medicines (eg, ferrous sulphate tablets and slow-release potassium chloride tablets for treating side-effects) and consumables were obtained from each facility involved in the IVON trial and the local pharmacies where drugs are sold at wholesale prices. We obtained ferric carboxymaltose cost estimates from three sources: the import cost during the IVON trial as the base-case estimate; a negotiated lower-limit price for bulk procurement for an ongoing separate ferric carboxymaltose project, adjusted to 2022 costs with the World Bank US GDP deflator;^21^ and an upper-limit cost from a public Indian pharmaceutical website (appendix 1 pp 3–5).

For both the ferric carboxymaltose and ferrous sulphate groups, we included costs of blood transfusion (covering blood screening, transfusion-related consumables, and skilled health-care personnel's time) and hospital admission for postpartum haemorrhage. Admission costs were based on WHO-CHOICE 2010 estimates for inpatient care at secondary-level facilities in Nigeria, adjusted to 2023 values with the World Bank US GDP deflator.^21^, ^23^ However, other direct medical costs related to both groups were not included as they would be the same for each group, including folic acid and intermittent preventive therapy, antenatal care booking, including consultation fees for each antenatal care and postnatal care visit, and cost for screening and diagnostic tests for anaemia in pregnancy (packed cell volume and full blood count).^4^ Additionally, facility costs for storing and distributing ferrous sulphate and ferric carboxymaltose were excluded from the model, as they are typically embedded within procurement prices and contribute minimally relative to overall drug acquisition and administration costs, making them unlikely to materially affect the results (appendix 1 pp 3–5).

In addition to direct medical costs, we included direct non-medical costs, namely transportation expenses for pregnant women attending antenatal care visits, both scheduled and unscheduled. We also considered indirect costs, which reflect productivity losses experienced by pregnant women and their caregivers. We used the human capital approach to estimate indirect costs associated with productivity loss. This estimate included time spent by pregnant women and their caregivers on health-related activities (eg, travel, waiting, or treatment) and time lost due to hospitalisation or unscheduled visits. Monetary value was assigned with the median hourly wage of participants (appendix 1 pp 3–5) and for unemployed participants, the Nigerian minimum wage (NGN 30 000 per month as of June 15, 2023) was used. No discount factor was used to account for future health-care costs beyond the event period, as the analytic horizon is less than a year. All costs were converted from Naira to US dollars by use of average temporal exchange rates applicable between Aug 10, 2021, and June 15, 2023 when data were collected. All cost estimates are presented in US dollars (appendix 1 pp 3–5).

### Health outcomes parameters

Model outcome parameters included in this analysis are described in table 1. Our model incorporates key clinical inputs from the treatment options, particularly focusing on persistent IDA at 36 weeks' gestation that can lead to complications such as postpartum haemorrhage and, in severe cases, death. We defined a favourable outcome as the resolution of IDA after treatment with either ferric carboxymaltose or ferrous sulphate. Conversely, an unfavourable outcome was described as the persistence of IDA, which can lead to complications and disabilities.^6^ For transition probabilities related to the development of complications, we gathered data from the IVON trial, relevant literature,^13^, ^14^, ^15^, ^24^, ^25^, ^26^ and expert opinion. We defined moderate postpartum haemorrhage as maternal haemorrhage of less than 1 L of blood loss within 24 h after birth and severe postpartum haemorrhage as blood loss of more than 1 L within 24 hours after birth, based on WHO guidelines.^29^ We also considered the probabilities and the effect of experiencing disability burden from treatment-related events, contributing to the terminal events of the clinical pathway in the model (table 1).^14^, ^15^, ^17^

**Table 1:** Model outcome parameters

	**Base-case values**	**Range (one-way sensitivity analysis)**	**Source**
**Ferric carboxymaltose**			
Probability of having persistent IDA	0·09	0·02–0·16	Afolabi et al (2024),^13^ Pasricha et al (2023),^14^ Breymann et al (2017)^15^
Probability of drug-related disability	0·08	0·02–0·11	Saha et al (2024),^17^ Breymann et al (2017),^15^ Pasricha et al (2023)^14^
**Ferrous sulphate**			
Probability of having persistent IDA	0·23	0·10–0·30	Afolabi et al (2024),^13^ Pasricha et al (2023),^14^ Breymann et al (2017)^15^
Probability of drug-related disability	0·26	0·03–0·60	Saha et al (2024),^17^ Breymann et al (2017),^15^ Pasricha et al (2023)^14^
**Both treatment options**			
Probability of postpartum haemorrhage from moderate anaemia	0·04	0·02–0·06	The WOMAN-2 Trial Collaborators (2023),^24^ Shi et al (2022),^25^ Nair et al (2021)^26^
Probability of maternal death with background moderate anaemia	0·000682	0·000545–0·000818	The WOMAN-2 Trial Collaborators (2023)^24^
Probability of maternal death with moderate anaemia and postpartum haemorrhage	0·016544	0·013235–0·019852	The WOMAN-2 Trial Collaborators (2023)^24^
**Disability values for both treatment options**			
Disability weight of moderate IDA	0·052	0·034–0·076	GBD 2019^27^
Disability weight of severe IDA	0·149	0·101–0·209	GBD 2019^27^
Disability weight of moderate postpartum haemorrhage	0·114	0·078–0·159	GBD 2019^27^
**Disability weight for adverse events**			
Ferric carboxymaltose; headache	0·037	0·022–0·057	GBD 2019^27^
Ferrous sulphate; diarrhoea	0·188	0·125–0·264	GBD 2019^27^
Life expectancy	53·38	48·68–58·14	WHO life tables by country^28^

Inconsistency in decimal places is due to data being from multiple sources and due to the rarity of some outcomes such as maternal death. GBD=Global Burden of Disease Study. IDA=iron deficiency anaemia.

For the cost-utility analysis, we estimated DALYs based on two important factors: (1) the incidence of non-fatal IDA events and (2) maternal mortality associated with IDA or non-IDA-related postpartum haemorrhage. For participants with non-fatal IDA, we calculated years lived with disability associated with persistent IDA, postpartum haemorrhage, and disability burden from treatment-related events. We used disability weights from the 2019 Global Burden of Disease Study to inform our analysis.^27^ In our model, the disability weight for severe IDA served as a proxy for persistent IDA, while the disability weight for moderate IDA was used for participants with resolved IDA (table 1). Each disability weight corresponded to estimated disability durations that were aligned with clinical course estimates of each health outcome based on the opinion of expert obstetricians (appendix 1 p 6). For cases of fatal IDA (used as a proxy for anaemia in the model), we assessed the years of life lost due to premature death with moderate anaemia and due to premature death with moderate anaemia and postpartum haemorrhage. Years of life lost was calculated by use of life expectancy figures specific to female populations in Nigeria, which were obtained from WHO's 2020 life expectancy tables, with the median age at first birth as a proxy for age at maternal death in Nigeria.^28^ DALYs were ultimately established by summing the years lost due to premature death and the years lived with disability.

### Model validation

Data quality was assessed using a graded approach that considered study design, relevance to the target population, and consistency of reported outcomes (table 1; appendix 1 p 6). Model calibration was done in a pragmatic manner by triangulating primary inputs against available literature and expert consensus to ensure clinical plausibility and relevance to real-world practice (appendix 1 p 6). For model validity, face and internal validation were conducted, including verification of the model structure and logical consistency, following established good practice guidelines for model validation by the International Society for Pharmacoeconomics and Outcomes Research Task Force (appendix 1 p 7).^30^ Key model inputs, where empirical data were unavailable, were based on expert-informed assumptions, as detailed in appendix 1 (p 6). Expert clinical input was further incorporated during model development to refine pathways and assumptions, with the effect of remaining uncertainty explored through sensitivity analyses (appendix 1 p 7).

### Statistical analysis

To calculate intervention cost, we aggregated direct and indirect costs across the groups. Where applicable, unit costs were multiplied by resource use estimates to calculate total costs per pregnant woman. We conducted a base-case analysis to estimate the relative cost-utility of ferric carboxymaltose compared with ferrous sulphate. Model outputs included the total expected cost and health outcomes per pregnant woman for each strategy. Health outcomes were measured as cases of DALYs averted. The incremental cost-utility ratio (ICUR) was calculated as the ratio of the difference in mean costs to the difference in mean effects between ferric carboxymaltose and ferrous sulphate:

$$\text{ICUR}=\frac{\text{mean}\;{\text{cost}}_{1}-\text{mean}\;{\text{cost}}_{2}}{\text{mean}\;{\text{DALYs}}_{1}-\text{mean}\;{\text{DALYs}}_{2}}$$

The ICUR was expressed as the additional cost per DALY averted. DALYs reflect health loss, with higher values indicating worse outcomes.^18^ To benchmark our ICUR, we used an opportunity cost-based cost-utility threshold based on Nigeria's average GDP per capita for 2021–22 (ie, $2078·35).^21^, ^31^ In the absence of a locally derived opportunity cost threshold for Nigeria, we followed the approach suggested by Ochalek and colleagues,^31^ who note that thresholds consistent with health opportunity costs in LMICs are typically below 1·0 times GDP per capita. On this basis, we applied a range of 0·5 to 1·0 times the GDP per capita ($1039·18–2078·35). Consequently, we classified ferric carboxymaltose as very cost-effective if the ratio was less than 0·5 times the GDP per capita and cost-effective if it fell between 0·5 and 1·0 times the GDP per capita.

Model performance was assessed through internal validation, including consistency and plausibility checks of the decision-tree logic (appendix 1 p 7). Where primary data were unavailable for specific parameters, values were sourced from comparable published studies or, where necessary, derived through expert elicitation (appendix 1 p 6). The effect of these data sources on the model's conclusions was further explored in the sensitivity analysis. We performed a one-way deterministic sensitivity analysis to identify key parameters influencing the outcome measures, including probabilities of persistent IDA for each intervention and probability of intervention-related disability. We included costs associated with drugs, including drug-related disabilities, transportation, and productivity losses for each treatment option, including disability weights and duration of IDA and persistent IDA associated with ferrous sulphate and ferric carboxymaltose. Parameter ranges were sourced from empirical data where available; otherwise, we used a 20% variation around the base-case values, as detailed in appendix 1 (pp 3–6). We presented our results in a tornado diagram highlighting the parameters with the greatest effect on ICUR.

We analysed the model using a Monte Carlo simulation with 1000 iterations to assess joint uncertainty across variables. We presented results using a scatter plot and cost-utility acceptability curve (CUAC). We based our analysis on distributions derived from the base-case (most likely), minimum, and maximum values. We applied a programme evaluation and review technique distribution for probabilities and a triangular distribution for costs, assuming the cost parameters vary by 20% from their most likely values. All analyses were done with TreeAge Pro 2025 R1 (TreeAge Software, Williamstown, MA, USA).

### Role of the funding source

The funder of the study had no role in study design, data collection, data analysis, data interpretation, or writing of the report.

## Results

Main results of the IVON trial have been reported previously.^19^ Here, we present the results of the cost-utility analysis. The total cost of ferric carboxymaltose for 527 pregnant women was $97 235·70, with a mean cost of $456·20 per pregnant woman. By contrast, the total cost of ferrous sulphate for 529 pregnant women was $76 665·58, with a mean cost of $427·34 per pregnant woman (table 2).

**Table 2:** Total costs and mean cost estimates per pregnant woman for ferric carboxymaltose and ferrous sulphate

	**Total costs, US$**	**Mean cost per pregnant woman, US$**
**Ferric carboxymaltose**		
Vials (n=527)*†	44 136·25	83·75
Administration (n=527)*	3262·13	6·19
Ferric carboxymaltose-related disability (n=9)‡	24·75	2·75
Total facility-based costs (n=527)*	2202·86	4·18
Postpartum haemorrhage (n=20)§	5592·00	279·60
Transport (n=527)*	10 666·48	20·24
Productivity (n=527)*	31 351·23	59·49
Total	97 235·70	456·20
**Ferrous sulphate**		
Drug course (n=529)*†	16 393·71	30·99
Administration (n=529)*	1010·39	1·91
Ferrous sulphate-related disability (n=15)‡	214·79	14·32
Postpartum haemorrhage (n=21)§	5871·60	279·60
Transport (n=529)*	13 002·82	24·58
Productivity (n=529)*	40 172·26	75·94
Total	76 665·58	427·34

* Total costs are the sum for the full cohort (n=527 for ferric carboxymaltose; n=529 for ferrous sulphate); mean costs are reported per pregnant woman.

† Ferric carboxymaltose is administered intravenously and procured in single-use vials; therefore, its cost was calculated per vial. By contrast, ferrous sulphate is an oral medication supplied in tablet form, and its cost was calculated per drug course.

‡ Treatment-related disability costs were incurred by a small number of participants (9 participants in the ferric carboxymaltose group; 15 participants in the ferrous sulphate group).

§ The number of participants with postpartum haemorrhage (n=20·45 for ferric carboxymaltose; n=20·53 for ferrous sulphate) was estimated by multiplying the average probability used in the model (0·0388) by the total number of women in each group.

In the base-case analysis, treatment with ferric carboxymaltose incurred a higher cost per pregnant woman ($139·71) than treatment with ferrous sulphate ($104·33), with a difference in incremental cost of $35·38 between the two treatments. Ferric carboxymaltose showed greater effectiveness by incurring lower DALYs (0·0111) than ferrous sulphate (0·0377), with an incremental effect of 0·0266 DALYs. The resulting ICUR was $1331·12 per DALY averted (table 3). This ICUR falls below the cost-utility threshold of $2078·35, which lies between 0·5 and 1·0 times GDP per capita; therfore, ferric carboxymaltose is considered cost-effective with the ICUR falling in the northeast quadrant (Q1) of the cost-effectiveness plane (ie, where the intervention is both more effective and more costly than the comparator).

**Table 3:** Expected cost, DALYS, and ICUR

	**Expected cost per pregnant woman, US$**	**DALYs incurred per pregnant woman**	**Incremental cost, US$**	**Incremental effect, DALYs**	**ICUR, US$**
Ferrous sulphate	104·33	0·0377	..	..	..
Ferric carboxymaltose	139·71	0·0111	35·38	0·0266	1331·12

DALY=disability-adjusted life-year. ICUR=incremental cost-utility ratio

**Figure 2:**
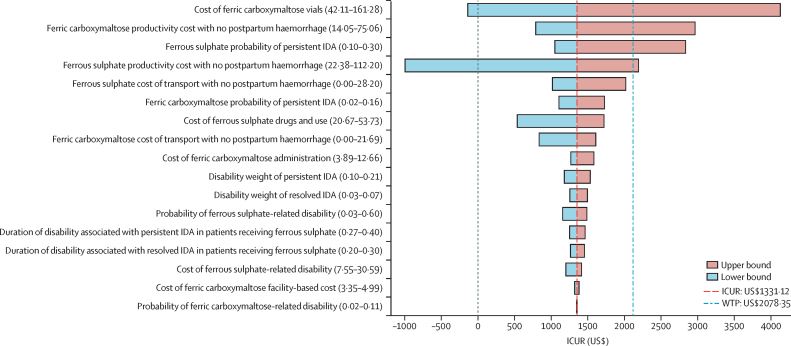
Tornado diagram showing the effect of varying each parameter on ICUR Numbers in parentheses are the parameter bounds. The dashed lines are the base-case ICUR and the WTP. The WTP line represents Nigeria's average gross domestic product per capita for 2021–22 ($2078·35). Threshold variable values are those that cross the WTP line. Persistent IDA refers to IDA that does not resolve after treatment (ie, treatment failure), whereas resolved IDA refers to anaemia that resolves following treatment (ie, treatment success). ICUR=incremental cost-utility ratio. IDA=iron deficiency anaemia. WTP=willingness to pay.

The one-way sensitivity analysis showed that the cost of ferric carboxymaltose vials is the most substantial factor influencing the ICUR. Of the total ICUR uncertainty (44 362 623), the cost of ferric carboxymaltose vials accounted for nearly half of the variation, contributing 45·3% (20 094 478), followed by the ferrous sulphate productivity cost without postpartum haemorrhage at 25·3% (11 212 164) and the ferric carboxymaltose productivity cost without postpartum haemorrhage at 11·8% (5 229 994; appendix 1 p 8). At a lower cost of ferric carboxymaltose vials ($42·11), the treatment is very cost-effective, with an ICUR of –$235·20, placing it below the cost-utility threshold (ie, less than 0·5 times GDP per capita). However, when the cost rises to $161·28, the ICUR increases to $4247·48, making it no longer cost-effective. The cost at which the ICUR exceeds the cost-utility threshold is US$103·61, at which point ferric carboxymaltose is not deemed cost-effective. Regarding ferrous sulphate productivity cost with no postpartum haemorrhage, we observed that at a lower productivity cost of $22·38, the ICUR increases to $2214·65, exceeding the cost-utility threshold, thereby making ferric carboxymaltose not cost-effective. Conversely, at a ferrous sulphate productivity cost with no postpartum haemorrhage of $112·20, the ICUR decreases to –$1133·80, making ferric carboxymaltose more cost-effective. The effect of the ferric carboxymaltose productivity cost without postpartum haemorrhage is smaller but still notable, with the ICUR ranging from $740·00 to $3026·91. At productivity costs above $49·75, the cost-effectiveness of ferric carboxymaltose is no longer assured. At a probability of 0·3 for persistent IDA after ferrous sulphate treatment, the ICUR decreases to $1015·32, which is below the cost-utility threshold. However, at a probability of 0·1, the ICUR rises to $2889·73, exceeding the threshold at a probability of 0·14. Further sensitivity analyses, exploring additional costs, probabilities, and DALYs for each treatment option, did not alter the base-case outcomes; the continued use of ferric carboxymaltose consistently showed its cost-effectiveness (figure 2).

**Figure 3:**
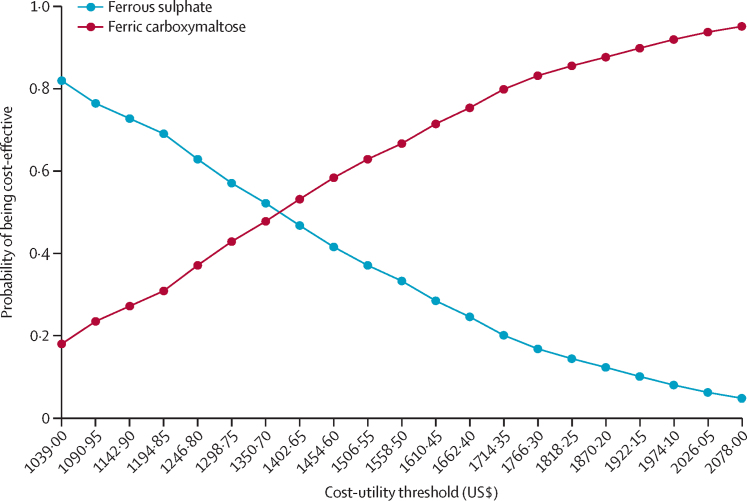
Cost-utility acceptability curve

From the scatter plot, ferric carboxymaltose was cost-effective compared with ferrous sulphate in most simulations, with the majority of ICUR values falling below the threshold (appendix 1 p 9). Specifically, of the 1000 cost-utility pairs, 956 (95·6%) are in Q1 of the cost-effectiveness plane below the threshold, while the remaining 44 (4·4%) lie above the threshold in the same quadrant (appendix 1 pp 9–10). Further analysis with the CUAC shows that ferric carboxymaltose has a 0·18 probability of being cost-effective at a threshold of $1039·18 per DALY averted, rising to 0·95 at $2078·35 (figure 3).

## Discussion

We evaluated the cost-utility of ferric carboxymaltose compared with ferrous sulphate for treating moderate-to-severe IDA during pregnancy in Nigeria using a decision-tree model. The expected cost per woman treated was higher for ferric carboxymaltose ($139·71) than for ferrous sulphate ($104·33). However, ferric carboxymaltose was more effective, resulting in fewer DALYs incurred per pregnant woman (0·0111 for ferric carboxymaltose *vs* 0·0377 for ferrous sulphate). The ICUR of ferric carboxymaltose was $1331·12 per DALY averted, which falls between 0·5 times and 1·0 times Nigeria's GDP per capita. This base-case finding shows that ferric carboxymaltose is a cost-effective intervention for managing moderate-to-severe IDA in pregnancy in Nigeria. This adds to the evidence base from earlier studies which established that a separate intravenous iron formulation, iron sucrose, which requires multiple administrations, is also more cost-effective than oral iron in general.^16^, ^17^ In our study, we used ferric carboxymaltose, a newer parenteral formulation which, despite its higher cost compared with iron sucrose, has the compelling benefit of requiring only a single treatment session, simplifying the treatment regimen and improving patient adherence.^12^

Our one-way sensitivity analysis showed that the cost of ferric carboxymaltose vials was the most influential driver of the ICUR, followed by its associated productivity costs. Ferric carboxymaltose remained cost-effective up to threshold values of $103·61 for ferric carboxymaltose vials and $49·75 for productivity losses. Beyond these threshold levels, ferric carboxymaltose is no longer cost-effective. As the primary cost driver, high cost of ferric carboxymaltose vials, often paid out of pocket could restrict access for the pregnant women who require it most.^2^ However, when the cost is reduced to $42·11, the lower limit of our base case, the ICUR drops below 0·5 times GDP per capita, making ferric carboxymaltose very cost-effective at this price point.

This study, which is the first economic evaluation comparing ferric carboxymaltose and ferrous sulphate for treating IDA in Africa, has several strengths. First, we adopted a partial societal perspective, incorporating both health-care and non-health costs. Second, we used a cost-utility analysis with DALYs, which is more commonly used for economic evaluations from LMICs, such as Nigeria.^18^ Third, we used a detailed microcosting approach, drawing most cost parameters directly from locally relevant sources. This approach enabled detailed and context-specific estimates of both direct and indirect costs.

Some limitations should be considered when interpreting our results. First, some of the data used in this study came from clinical trials and, although pragmatic, such data limit our understanding of ferrous sulphate-related persistent IDA probabilities in real-world scenarios. In routine practice, many pregnant women initiate antenatal care late, limiting early diagnosis and timely treatment, particularly with ferrous sulphate. This delay is compounded by higher non-adherence rates for ferrous sulphate, resulting in more treatment failures and persistent IDA than our model suggests.^5^ Thus, the effectiveness of ferrous sulphate in our model might be overestimated relative to what would typically be observed in routine clinical practice, suggesting that ferric carboxymaltose might be even more cost-effective in comparison. Second, the short time horizon, restricted to pregnancy and the postpartum period, might have excluded longer-term maternal consequences of IDA and underestimated associated costs and health effects, as robust data were unavailable to support extrapolation beyond the trial. However, this time horizon avoids the introduction of additional structural model uncertainty, which could have arisen from unsupported long-term assumptions a similar consideration made in a previous economic evaluation.^16^ Third, this economic evaluation was done based on established clinical effectiveness for maternal outcomes, providing the necessary prima facie justification for modelling. Accordingly, the analysis focused exclusively on maternal outcomes and did not include potential neonatal benefits, which might have led to an underestimation of health gain. However, existing trial evidence has not shown neonatal effects of sufficient magnitude to influence the results.^13^, ^14^ Moreover, inclusion of any potential neonatal benefits would only be expected to further improve the cost-effectiveness of ferric carboxymaltose relative to ferrous sulphate, as ferric carboxymaltose has been shown to enable faster and more complete correction of IDA. This reduction in IDA would likely translate into greater reductions in adverse neonatal outcomes compared to oral iron. As a result, inclusion of neonatal benefits would increase the incremental effectiveness of ferric carboxymaltose without a proportional increase in cost, thereby reducing the ICUR. Finally, although opportunity-cost estimates for Nigeria from Ochalek and colleagues^31^ suggest substantially lower thresholds, akin to less than 0·5 times GDP per capita, their more recent work shows that single GDP-based thresholds can misrepresent (ie, underestimate or overestimate) true opportunity costs in many LMICs.^32^ Therefore, to avoid prematurely dismissing interventions that could still deliver value, particularly in maternal health, we adopted a pragmatic threshold of 0·5–1·0 times GDP per capita, while recognising that scarce locally derived data might constrain accuracy.^32^

There are important practice, policy, and research implications emerging from our findings. For practice, skilled health-care personnel should consider ferric carboxymaltose as a cost-effective treatment option to rapidly optimise the haemoglobin concentrations of pregnant women with IDA as part of antenatal care. Existing evidence showed that ferric carboxymaltose is an acceptable treatment option for both pregnant women and skilled health-care personnel.^10^ Policy instruments can be used to stimulate its use in practice. To start, policy makers and service planners need to ensure that ferric carboxymaltose is integrated into national treatment guidelines for managing moderate-to-severe IDA in pregnancy, with a clear clinical pathway for its use. This pathway should align with the realities of antenatal care service delivery in each country. For countries where IDA is highly prevalent, ferric carboxymaltose should be considered for women diagnosed with moderate-to-severe asymptomatic anaemia after routine haemoglobin screening, or after confirmatory ferritin testing, where laboratory capacity permits. This approach layers feasibility considerations on top of cost-effectiveness,^33^ ensuring timely treatment while optimising resource use, and acknowledges potential drawbacks such as the risk of overtreating pregnant women without IDA and inequities where skilled health-care personnel capacity is scarce. Our study provides robust evidence supporting the integration of ferric carboxymaltose into clinical practice, clearly indicating that allocating resources to ferric carboxymaltose offers better value than to ferrous sulphate. This evidence is particularly important in populations where treatment failure leads to long-term health and productivity costs.^5^ In addition, the routinisation of ferric carboxymaltose use among pregnant women in LMICs, such as Nigeria, hinges largely on its affordability. Indeed, the high price of ferric carboxymaltose can translate into considerable out-of-pocket expenses for pregnant women and their families, which risks creating substantial financial burden and access inequities.^10^, ^34^ As such, minimising price at the point of purchase will be crucial if uptake is to be realised. Substantial uptake can be reached through the implementation of upstream strategies such as government negotiations for lower wholesale prices of the medication and streamlined logistics through bulk shipping for imported ferric carboxymaltose vials combined with stimulation of local production. The Nigerian Government's recent policy of exempting pharmaceutical products and medical devices from value added tax could further lower costs if extended to ferric carboxymaltose.^35^ Furthermore, to ensure universal and equitable access to ferric carboxymaltose, the medication should be included in the list of medications accessible through health insurance schemes, which will minimise the financial burden to pregnant women and their families. Future research should prioritise DALY-based assessments, given their wider application in LMICs and the need for greater comparability across studies. There is also a need to consider the comparative cost-effectiveness of ferric carboxymaltose and iron sucrose, as ferric carboxymaltose remains the more expensive formulation despite the clinical advantage of single-dose administration.^12^, ^13^

In conclusion, ferric carboxymaltose is a cost-effective intervention to reduce the maternal morbidity burden from moderate-to-severe IDA in Nigeria. Integration into national guidelines for antenatal care and health insurance schemes based on negotiated prices, especially in high-burden settings, will help advance health equity and maximise outcome gains for pregnant women and the wider health system.

### Contributors

### Equitable Partnership Declaration

### Data sharing

Data used in this study were obtained from publicly available sources, all of which are listed in table 1 and appendix 1 (pp 3–6).

## Declaration of interests

We declare no competing interests.

## Supplementary Materials

Supplementary appendix 1

Equitable Partnership Declaration
