# Supplementary material for: Intravenous ferric carboxymaltose versus oral ferrous sulphate for iron deficiency anaemia in pregnancy in Nigeria: a cost-utility analysis
Source: Lancet Obstet Gynaecol Womens Health. 2026 Jun;2(6):e524–34. doi: 10.1016/S3050-5038(26)00049-X (PMC13288457; doi:10.1016/S3050-5038(26)00049-X)
Supplement: Supplementary appendix 1 [file mmc1.pdf]

THE LANCET
Obstetrics, Gynaecology, & Women's Health

Supplementary appendix 1

This appendix formed part of the original submission and has been peer reviewed. We post it as supplied by the authors.

Supplement to: Akinajo OR, Annerstedt KS, Santos MT, Afolabi BB, Banke-Thomas A. Intravenous ferric carboxymaltose versus oral ferrous sulphate for iron deficiency anaemia in pregnancy in Nigeria: a cost-utility analysis. *Lancet Obstet Gynaecol Womens Health* 2026; published online April 28. https://doi.org/10.1016/S3050-5038(26)00049-X.

Supplementary appendix 1

Supplement to: Opeyemi R **Akinajo**, Kristi Sidney **Annerstedt**, Maria Teresa **Santos**, Bosede Bukola **Afolabi**, Aduragbemi **Banke-Thomas**. Intravenous ferric carboxymaltose versus oral ferrous sulphate for iron deficiency anaemia in pregnancy in Nigeria: a cost-utility modelling analysis. *The Lancet Obstetrics, Gynaecology, & Women's Health.* 2026 doi: thelancetobgynwh-D-0745R2S3050-5038(26)00049-X

Table of Contents

Supplementary Methods ... *1*

Figure S1. Map of Nigeria highlighting Kano and Lagos states with characteristics of healthcare facilities where the study was conducted ... 1

Additional details on costing methods ... 2

Table S1. Model cost parameters ... 3

Table S2. Key model assumptions on disability duration for health outcomes ... 6

Validation method ... 7

Supplementary Results ... *8*

Table S3. One-way sensitivity analysis data underlying Figure 2 ... 8

Figure S3. Scatter plot of incremental cost (in dollars) and effectiveness of Ferric Carboxymaltose versus Ferric sulphate ... 9

Table S4. Scatter plot report: Incremental cost-utility analysis of FCM versus FS ... 10

References ... *11*

Supplementary Methods

Figure S1. Map of Nigeria highlighting Kano and Lagos states with characteristics of healthcare facilities where the study was conducted

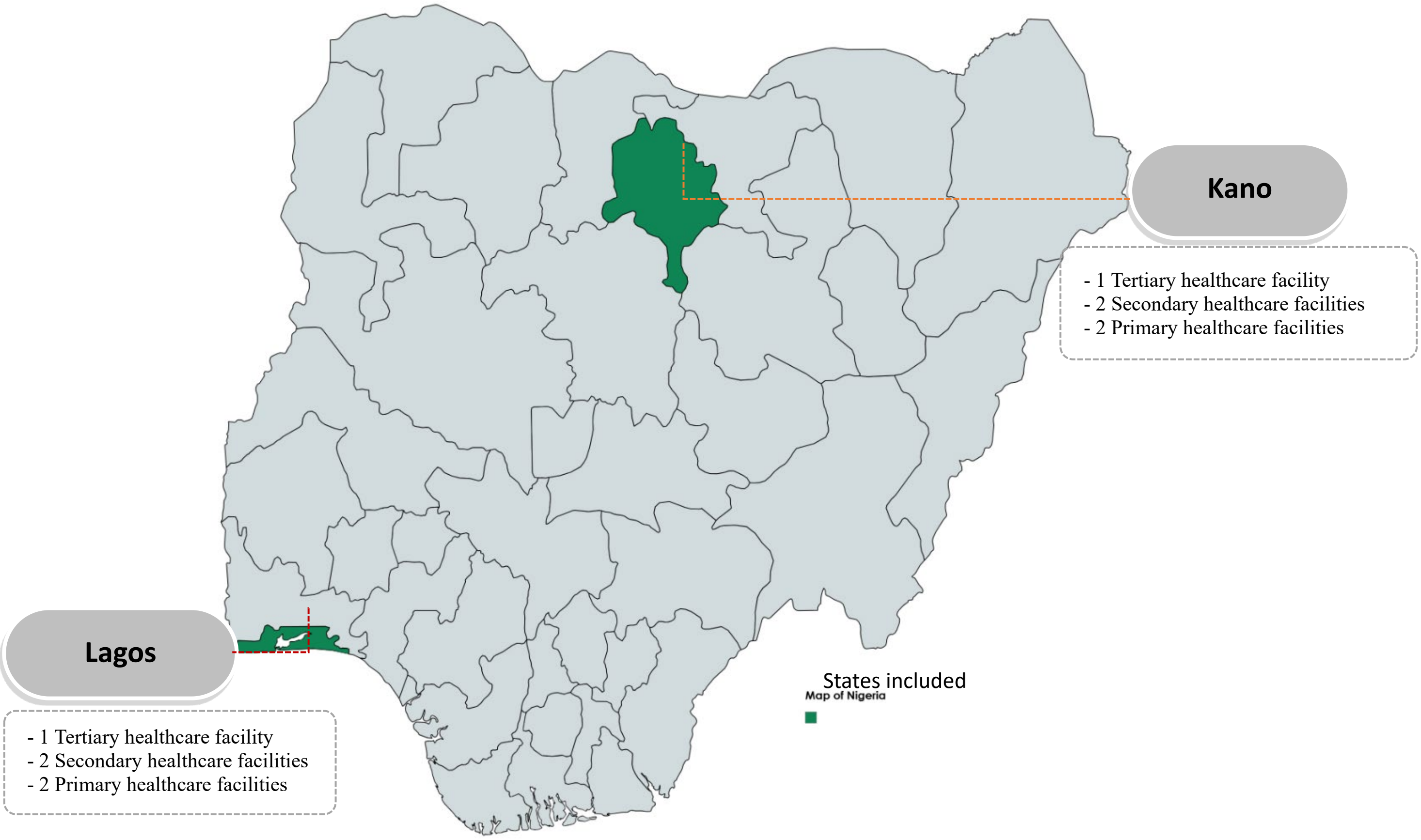

**Primary, secondary, and tertiary constitute the three levels of the healthcare system in Nigeria. The number of facilities selected at each level in each state is included in the box.*[1]

Additional details on costing methods

The costs were categorised into direct medical, direct non-medical, and indirect costs. Under the direct costs, we have user cost, SHPs' cost, and hospital cost, as detailed in Table S1.

For user cost, we calculated the costs associated with each FCM administration by considering several factors. First, we included the cost of each FCM vial, including shipping, customs duties, clearance, and distribution in Nigeria. We derived FCM cost estimates from three sources: a base-case estimate from a supplier during the IVON trial, a negotiated lower-limit price for bulk procurement from an ongoing separate FCM project adjusted to 2022 costs using the World Bank's US GDP deflator, and an upper-limit cost from a public Indian pharmaceutical website. [2–4] We also calculated the costs of consumables essential for FCM administration, including emergency readiness measures required during FCM administration, as emergency consumables [Table S1].

For FS consumption, assuming all pregnant women in the IVON trial began ANC in the first trimester, each woman used 200 mg of FS three times daily for 245 days (203 days during pregnancy and 42 days postpartum). Cost data for FS were obtained from trial facilities and local pharmacies where drugs are sold at wholesale prices. Over 50% of pregnant women experience adherence issues to FS, primarily due to gastrointestinal drug-related disabilities.[5] We based our analysis on common drug-related disabilities reported in the IVON trial, such as diarrhoea for FS and headaches for FCM. To manage FS-related disabilities classified as moderate, we assumed that unscheduled visits, both initial and follow-up, to the ANC would be necessary, including laboratory investigations. Additionally, we included the costs of medications used to manage drug-related disabilities, such as oral rehydration solutions, oral potassium tablets and consumables used to manage the drug-related disabilities [Table S1].

For both treatment options, we included the management of PPH resulting from persistent IDA. We estimated the average hospital stay to be three days, with a plus or minus one-day range for the lower and upper limits. Admission costs were based on WHO-CHOICE 2010 estimates for secondary-level facilities in Nigeria, using the distribution of FCM administration in the IVON trial, which is highest in secondary healthcare facilities.[6,7] These estimates were adjusted to 2023 values using the US GDP deflator.[2] We assumed each pregnant woman required two units of blood to manage PPH [Table S1].

For SHP cost, we used data from each facility's records to estimate salaries by calculating hourly wage rates and multiplying them by the time spent on each activity.[8] We started by calculating the time required for SHPs to administer FCM, which ranges from 50 to 60 minutes, and valued this time using the median salary of the lowest cadre of SHPs based on their hourly activity. For SHP time to prescribe and dispense FS, we accounted for the time doctors take to prescribe FS (1–3 minutes) and the time pharmacists take to dispense and instruct pregnant women (5–10 minutes). Additionally, we factored in the time spent by SHPs to manage drug-related disabilities for both treatment options and related tasks during patient visits. Furthermore, we accounted for SHP time to manage PPH. We factored in the time SHPs, particularly nurses, spend on blood transfusions using a partial engagement approach that reflects real-world clinical settings where nurses typically manage multiple patients simultaneously. Although it takes about four hours to transfuse each unit of blood, totalling eight hours for two units, we assumed that the nurse is actively engaged for only 50% of this time, equating to four hours. This time covers setup, intermittent monitoring, and follow-up, helping avoid overestimating SHP's time. For the sensitivity analysis, we used 25% of the total time (two hours) as the lower limit and the full eight hours as the upper limit [Table S1].

For hospital costs, we included the capital costs of monitoring and infusion equipment required for service delivery. Monitoring equipment, including a blood pressure apparatus, pulse oximeter, and drip stand, as well as emergency equipment such as an oxygen cylinder that must always be on standby in case of any FCM-related disabilities during administration, was treated as capital costs and annualised over its useful life using a 3% discount rate, with uncertainty in costs and useful life years explored in sensitivity analysis.[9] To reflect routine service delivery rather than trial utilisation, annualised costs were allocated on a per-patient basis. Annual facility deliveries were used as a proxy for the size of the ANC population (3,600 deliveries/year).[10] A routine FCM uptake of 15% was assumed. The resulting per-patient costs were aggregated across devices to derive a single monitoring cost, which was applied to the FCM arm only and varied in sensitivity analyses [Table S1].

For direct non-medical costs, we included transportation costs for pregnant women travelling to the hospital for ANC visits, including unscheduled visits in the FS group and admissions for PPH management in both groups. We used the transportation costs from each woman's final visit in the IVON trial as a proxy for the costs of all visits. While ANC initiation varied among participants, all attended at least one visit. We assumed that the costs from the final visit also represented those from previous visits. In the FCM group, transportation costs were multiplied by 11, reflecting the expected number of scheduled ANC visits and PPH management. For the FS group, we multiplied the transportation costs by 13 to account for both scheduled and unscheduled visits related to FS disabilities and PPH management. For sensitivity analysis, we set a lower limit of $0 for participants with no expenses and an upper limit 50% above the base estimate to account for extreme cases.

Lastly, we accounted for indirect costs associated with productivity losses among pregnant women and their caregivers using the Human Capital Approach.[9,11] We included the time spent on health-related activities, such as travel, waiting, and treatment, as well as any time lost due to unscheduled visits or hospitalisation. We assigned a monetary value to this time using the median hourly wage, and for participants who were unemployed, we used the Nigerian minimum wage as a proxy. In the sensitivity analysis, we set the lower limit at the minimum wage and the upper limit at the 95th percentile of women's salaries to manage outliers. No discount factor was applied for future healthcare costs beyond the event period, as our analytic horizon is less than a year. All unit costs were converted from Naira to U.S. dollars using the average exchange rates for the period from 2021 to 2023.

Table S1. Model cost parameters

				Naira rate per unit cost				Average dollar rate per quantity			
Categories	Name	Resource use	Unit cost	Reference case	Lower bound	Upper bound	Quantity	Reference case	Lower bound	Upper bound	Source
**Direct Medical costs**											
	FCM administration	FCM	Per vial	17375.52	8736.51	33510.37	2 vials	83.75	42.11	161.51	IVON trial, *CAPREMAN, **IndiaMart
	Consumables	Gloves	Per pair	55.5	48	70	1 pair	0.13	0.12	0.17	Facilities
		Syringe and needle	Per 1	50	25	100	1	0.12	0.06	0.24	Facilities
		Cotton wool	Per 1	110	100	600	1	0.27	0.24	1.45	Facilities
		Plaster	Per 1	400	50	700	1	0.96	0.12	1.69	Facilities
		Methylated spirit	Per 1	200	150	300	1	0.48	0.36	0.72	Facilities
		Fluid giving set	Per 1	150	100	250	1	0.36	0.24	0.60	Facilities
		Normal saline	Per 1	607.5	550	1200	1	1.46	1.33	2.89	Facilities
		Cannula	Per 1	100	80	150	1	0.24	0.19	0.36	Facilities
	Emergency consumables	Adrenaline	Per 1	200	160	240	1	0.482	0.386	0.578	Facilities
		Oxygen mask	Per 1	1500	1200	1800	1	3.615	2.892	4.338	Facilities
	FS tablets	Fesolate	Per pack	1500	1000	2700	7.35 packs	26.57	17.71	47.82	Facilities
	FS tablets use	Water	Per sachet	7.5	5	10	1 daily * 245 days	4.42	2.95	5.90	Market price
	FS drug-related disabilities treatment (medications, consumables and investigation)	ORS	Per sachet	66.5	33	120	4,3,5 respectively	0.64	0.24	1.45	Facilities
		Tabs slow K	for 2 sachets	719	150	1288	2 sachets	3.47	0.72	6.21	Facilities
		Gloves	Per pair	55.5	48	70	1 pair	0.13	0.12	0.17	Facilities
User costs		Syringe and needle	Per 1	50	25	100	1	0.12	0.06	0.24	Facilities
		Cotton wool (mini pack)	Per 1	110	100	600	1	0.27	0.24	1.45	Facilities
		Methylated spirit	Per 1	200	150	300	1	0.48	0.36	0.72	Facilities
		Electrolyte, urea and creatinine	Per analysis	2500	2000	5000	1 analyte	6.03	4.82	12.05	Facilities
		Blood screening	Per screening	7000	500	10500	2 units	33.74	1.21	75.92	Facilities
		Gloves	Per pack	2775	2400	3500	1 pack	6.69	5.78	8.44	Facilities
		Syringes and needles	Per 1	50	25	100	5,4,6 respectively	0.60	0.24	1.45	Facilities
	Management of PPH (Blood transfusion and consumables and hospital admission)	Cotton wool (mini pack)	Per 1	110	100	600	1	0.27	0.24	1.45	Facilities
		Plaster	Per 1	400	50	700	1	0.96	0.12	1.69	Facilities
		Methylated spirit	Per 1	200	150	300	1	0.48	0.36	0.72	Facilities
		Fluid giving set	Per 1	150	100	250	1	0.36	0.24	0.60	Facilities
		Intravenous fluid	Per 1	607.5	550	1200	6,5, and 7 respectively	8.78	6.63	20.24	Facilities
		Cannula	Per 1	100	80	150	2	0.48	0.19	1.08	Facilities
		Length of stay on admission	Secondary facility	30165	11282	64190	3, 2 and 4 days respectively	218.09	54.38	618.79	Facilities
SHPs' costs	SHP FCM administration-related time	Prescription time	Medical officer income per hour	1443	1121	1800	2,1 and 3 minutes * 1	0.12	0.05	0.22	Facilities
		Dispensing time	Pharmacist income per hour	880	424	1369	7.5, 5 and 10 minutes *1	0.27	0.09	0.55	Facilities

		Preparatory/ administration/observation time	Nursing officer income per hour	768	549	1445	57.5, 50 and 65 minutes *1	1.77	1.10	3.77	Facilities
	SHP time to treat drug related disabilities to FCM	Observation time	Nursing officer income per hour	768	549	1445	1 hour, 30 minutes and 2 hours *1	2.31	0.66	6.96	Facilities
		Review of drug-related disabilities	Medical officer income per hour	1443	1121	1800	7.5, 5 and 10 minutes *1	0.43	0.23	0.72	Facilities
	SHP time to prescribe and dispense FS	Prescription time	Medical officer income per hour	1443	1121	1800	2, 1 and3 minutes * 1	0.58	0.18	1.30	Facilities
		Dispensing time	Pharmacist income per hour	880	424	1369	7.5, 5 and 10 minutes *1	1.33	0.34	3.29	Facilities
	SHP time to treat drug- related disabilities to FS	Unscheduled review	Nursing officer income per hour	768	549	1445	7.5,5 and 10 minutes *2,1,3 visits	0.46	0.11	1.74	Facilities
		Unscheduled review	Medical officer income per hour	1443	1121	1800	15,10 and 20 minutes *2,1,3 visits	1.74	0.45	4.34	Facilities
		Dispensing time	Pharmacist income per hour	880	424	1369	7.7, 5 and 10 minutes * 1 visit	0.2651	0.09	0.55	Facilities
	SHP time to manage PPH	Analysis time (investigation)	Laboratory scientist income per hour	717	423	1396	25, 20 and 30 minutes * 1 visit	0.72	0.34	1.68	Facilities
		Grouping and crossmatching of blood	Laboratory scientist income per hour	717	423	1396	60, 48 and 72 mins *1	1.73	0.82	4.04	Facilities
		Blood transfusion	Nursing officer income per hour	768	549	1445	4,2 and 8 hours * 1	7.40	2.65	27.86	Facilities
Hospital costs	Equipment	Sphygmomanometer	Useful life of 6,5 and 7years	7811.56	7014.7	6643.67	540	0.035	0.031	0.030	***Facilities/Literature
		Pulse oximeter	Useful life of 6,5 and 7years	1336.19	1199.88	1136.42	540	0.006	0.0053	0.0051	
		Oxygen cylinder	Useful life of 6,5 and 7years	8346.33	7619.98	7334.30	540	0.037	0.034	0.032	
		Drip stand	Useful life of 6,5 and 7years	1258.56	1139.65	1088.22	540	0.006	0.005	0.004	
**Direct non-medical costs**											
**Users' costs**	FCM	Transport cost per visits	Round trip per visit	600	0	900	11 visits	15.91	0	23.86	IVON Trial
	FS	Transport cost per visits	Round trip per visit	600	0	900	13 visits	18.79	0	28.19	IVON Trial
**Care givers' costs**	FCM (admission for PPH management)	Transport cost per visits	Round trip per visit	600	0	900	3 visits	4.34	0	6.51	IVON Trial
	FS (admission for drug-related disabilities and PPH management)	Transport cost per visits	Round trip per visit	600	0	900	4 visits	5.78	0	8.68	IVON Trial
**In-direct costs**								**Time lost in hours**			
**Users' costs**	FCM related	User working time	Working hours per day, week and month	-	-	-	-	8 hours	40 hours	160 hours	IVON Trial

		Time spent to travel (round trip)	Round trip per visit in minutes	60	40	90	Time travel *11 visits	11	7.33	16.5	IVON Trial
		User waiting time to see the SHP	Waiting time in minutes	162.8	137.6	188	Waiting time * 10 visits	27.13	22.93	31.33	IVON Trial
		User waiting time to receive FCM at the pharmacy	Waiting time in minutes	15	10	25	Waiting time * 1 visit	0.25	0.17	0.42	IVON Trial
		User time for FCM administration	Administration time in minutes	57.5	50	65	Administration time *1 visit	0.96	0.83	1.08	IVON Trial
		Extra time for observation (drug-related disabilities)	Observation time in minutes	75	30	120	Observation time * 1 visit	1.25	0.5	2	IVON Trial
		User time off work (PPH)	Time in hours	-	-	-	-	24	19.2	28.8	IVON Trial
		User income	Income per month	50000	30000	100000	income per hour	0.75	0.45	1.51	IVON Trial
		User working time	Working hours per day, week and month	-	-	-	-	8	40	160	IVON Trial
		Time spent to travel (round trip) for ANC visits, drug-related disabilities and PPH	Round trip per visit in minutes	60	40	90	Time travel *13 visits	13	8.67	19.5	IVON Trial
		User waiting time to see the SHP	Waiting time in minutes	162.8	137.6	188	Waiting time * 10 visits	27.13	22.93	31.33	IVON Trial
	FS related	User waiting time to receive FS at the pharmacy	Waiting time in minutes	15	10	25	Waiting time * 6,5 and 7 visits	1.5	0.83	2.91	IVON Trial
		User time off work (drug-related disabilities)	Time in hours	-	-	-	Working hours off work	16	12.8	19.2	IVON Trial
		User time off work (PPH)	Time in hours	-	-	-	Working hours off work	24	19.2	28.8	IVON Trial
		User income	Income per month	50000	30000	100000	income per hour	0.75	0.45	1.51	IVON Trial
		Working time	Working hours per day, week and month	-	-	-	-	8	40	160	IVON Trial
	FCM related	Total caring time	Time in hours	-	-	-	Working hours off work	24	19.2	28.8	IVON Trial
		Care giver income	Income per month	30000	24000	36000	Income per hour	0.45	0.36	0.54	IVON Trial
**Caregivers' costs**		Working time	Working hours per day, week and month	-	-	-	-	8	40	160	IVON Trial
	FS related	Caring time (drug-related disabilities)	Time in hours	-	-	-	Working hours off work	8	6.4	9.6	IVON Trial
		Caring time (PPH)	Time in hours	-	-	-	Working hours off work	24	19.2	28.8	IVON Trial
		Care giver income	Income per month	30000	24000	36000	Income per hour	0.45	0.36	0.54	IVON Trial

FCM, Ferric Carboxymaltose, FS, Ferrous Sulphate, SHP, Skilled Healthcare Personnel, *PPH, Post Partum Haemorrhage, IVON trial, Intravenous versus Oral Iron for Iron Deficiency Anaemia in Pregnant Nigerian Women, CAPREMAN, Comprehensive Approach to the Prevention and Management of Maternal Anaemia in Nigeria*

**CAPREMAN is an ongoing separate project on using FCM to manage anaemia in Pregnancy[3]*

*** IndiaMart is a public Indian pharmaceutical website[4]*

****Equipment was treated as capital costs, assuming 3600 deliveries per year and 15% annual uptake of FCM[10]*

Table S2. Key model assumptions on disability duration for health outcomes

Parameters	Assumption	Justification	Source
**FS-treatment related disability duration**			
Diarrhoea	0.0082 (0.0065 -0.0098)	In the IVON trial, diarrhoea emerged as the most prevalent FS-treatment related disability associated with FS. Consequently, we structured our model around this disability. For participants to have reported diarrhoea as a noticeable and treatment-related burden and typically required medication on an outpatient basis, but not hospitalisation, we considered it moderate in severity. This classification aligns with GBD criteria, which associate outpatient-managed symptoms that limit daily activities with moderate disability. We applied a disability weight of 0.188 accordingly. We assumed a disability duration of 72 hours (0.0082 years), with a range of 57.6 to 86.4 hours (±20 %) based on expert opinion.	IVON trial [12] Expert opinion
**FCM-treatment related disability duration**			
Headache	0.0027 (0.0022 - 0.0033)	One of the most common treatment-related disabilities associated with FCM is headache. We used symptomatic tension-type headaches as a proxy for those resulting from FCM treatment. Since most of these headaches are generally mild and self-limiting, we assumed a disability duration of 24 hours (0.0027 years) for our base case. We also considered a possible range of 19.2 to 28.8 hours, reflecting a variation of ±20%. These assumptions were based on expert opinion.	IVON trial [12] [13] Expert opinion
**FS-treatment related disability duration**			
Resolved IDA	0.25 (0.16666 - 0.3333)	Adherence to the recommended use of FS is often poor in real-life settings, typically resulting in longer treatment durations. However, since we lack data on the typical duration required to correct IDA in routine practice, we assumed a conservative duration, i.e. three months for resolved IDA with a range of two to four months and four months for cases of persistent IDA with a range of three to five months for lower and upper limits. These assumptions were based on expert opinion.	[14] Expert opinion
Persistent IDA	0.3333 (0.25- 0.4166)		
**FCM-related disability duration**			
Resolved IDA	0.08 (0.0577 - 0.0962)	For FCM, studies have shown that it rapidly corrects IDA and replenishes previously depleted iron within 4-6 weeks; therefore, we assumed a disability duration of 4 weeks for resolved IDA (3 and 5 weeks) and a disability duration of 5 weeks for persistent IDA (4 and 6 weeks).	[15–17]
Persistent IDA	0.10 (0.0769 – 0.1153)		
PPH disability duration	0.0191 (0.0109 - 0.0273)	For PPH, we applied a disability duration of 7 days as the base case, with lower and upper limits of 4 days and 10 days, respectively, reflecting a ±3 variation from the base case.	[18]

FS, Ferrous Sulphate, FCM, Ferric Carboxymaltose, IDA, Iron Deficiency Anaemia, PPH, Post Partum Haemorrhage

Validation method

The model was developed using a structured, pragmatic approach aligned with established good research practices for health economic modelling. We first assessed the quality of available clinical and economic data using a graded framework, evaluating sources based on study design, relevance to the target population, and consistency of reported outcomes. Where empirical data were incomplete or unavailable, key parameters were informed by expert opinion. These inputs were iteratively refined through comparisons with values reported in the broader literature, ensuring that the baseline model parameters were plausible and highly representative of current real-world clinical practice.

Model robustness was subsequently assessed through a rigorous combination of face and internal validation. **Face validity** was established by reviewing the model's conceptual structure, clinical pathways, and underlying assumptions with subject-matter experts to ensure strict alignment with current clinical understanding and treatment guidelines. **Internal validation** focused on the mathematical and logical integrity of the model. This process checks the accuracy of model calculations and verifies the logical consistency of the decision tree. Furthermore, hand calculations were performed for selected patient pathways to confirm that the model functioned exactly as intended.[19]

Beyond internal mechanics, the model's macroscopic outputs were evaluated for clinical plausibility. Given the limitations and scarcity of independent real-world datasets, a formal predictive external validation was not feasible. Instead, we employed a pragmatic cross-validation approach. Model outputs were benchmarked against published epidemiological evidence and patterns observed in real-world settings. These comparisons served as a vital reality check to ensure that the model's synthesized outputs remained directionally and proportionally consistent with known disease behaviour.[19]

Finally, structural and parameter uncertainty within the model was explored through comprehensive sensitivity analyses. The insights generated from these analyses were used formatively; by identifying which parameters drove the most variance in the outcomes, we were able to further refine sensitive model assumptions and pathways, ultimately yielding a conservative and clinically defensible evaluation.

Supplementary Results

Table S3. One-way sensitivity analysis data underlying Figure 2

Variable Name	Variable Description	Variable Low	Variable Base	Variable High	Impact	Low	High	Spread	Spread²	Risk %	Cum Risk %	Threshold Variable Value
Civ	Cost of FCM vials	42.11	83.7500	161.28	Increase	-235.2035225	4247.482989	4482.686511	20094478.36	0.452959655	0.452959655	103.6146742
Cprod_nopph_o	FS productivity cost with no PPH	22.38	46.08	112.2	Decrease	-1133.806873	2214.650077	3348.456951	11212163.95	0.252738977	0.705698632	26.03615361
Cprod_nopph_iv	FCM productivity cost with no PPH	14.05	29.82	75.06	Increase	739.995011	3026.9132	2286.918189	5229994.802	0.11789192	0.823590552	49.75439829
Probper_ida_o	FS probability of persistent IDA	0.1	0.23	0.3	Decrease	1015.32146	2889.732825	1874.411365	3513417.964	0.079197706	0.902788258	0.146076501
Co	Cost of FS drugs and use	20.67	31	53.73	Decrease	476.1133409	1719.694914	1243.581573	1546495.129	0.034860318	0.937648576	
Ctrans_nopph_o	FS cost of transport with no PPH	0	18.8	28.2	Decrease	980.6941091	2031.979859	1051.285749	1105201.727	0.024912903	0.962561479	
Ctrans_nopph_iv	FCM cost of transport with no PPH	0	14.46	21.69	Increase	789.0994664	1602.134305	813.0348388	661025.6491	0.014900509	0.977461988	
Probper_ida_iv	FCM probability of persistent IDA	0.02	0.09	0.16	Increase	1073.341192	1726.058585	652.7173935	426039.9958	0.00960358	0.987065568	
DWper_ida	Disability weight of persistent IDA	0.1	0.149	0.21	Decrease	1151.633665	1521.623591	369.9899257	136892.5452	0.003085763	0.990151331	
Prob_ao	Probability of FS-related disability	0.03	0.26	0.6	Decrease	1127.365254	1473.23766	345.8724058	119627.7211	0.002696588	0.992847919	
Cadmin_iv	Cost of FCM administration	3.89	6.19	12.66	Increase	1244.606128	1574.497549	329.8914215	108828.35	0.002453154	0.995301073	
DW_ida	Disability weight of IDA	0.03	0.052	0.07	Decrease	1229.403806	1480.875805	251.471999	63238.16628	0.001425483	0.996726556	
C_ao	Cost of FS-related disability	7.55	14.3196175	30.59	Decrease	1171.996182	1397.330454	225.3342727	50775.53445	0.001144557	0.997871113	
DDper_ida_o	FS-disability duration of persistent IDA	0.27	0.333333333	0.4	Decrease	1225.905442	1449.293417	223.3879743	49902.18707	0.00112487	0.998995983	
DD_ida_o	FS-disability duration of IDA	0.2	0.25	0.3	Decrease	1237.900115	1439.529369	201.6292542	40654.35614	0.00091641	0.999912393	
Cfac_based_iv	Cost of FCM facility-based cost	3.352	4.180692346	4.989	Increase	1299.950686	1361.52791	61.57722429	3791.754551	8.54718E-05	0.999997865	
Prob_aiv	Probability of FCM-related disability	0.02	0.076666667	0.11	Increase	1324.995612	1334.728019	9.732406813	94.71974238	2.13512E-06	1	

FCM, Ferric Carboxymaltose, FS, Ferrous Sulphate, PPH, Post Partum Haemorrhage, IDA, Iron Deficiency Anaemia

Figure S3. Scatter plot of incremental cost (in dollars) and effectiveness of Ferric Carboxymaltose versus Ferric sulphate

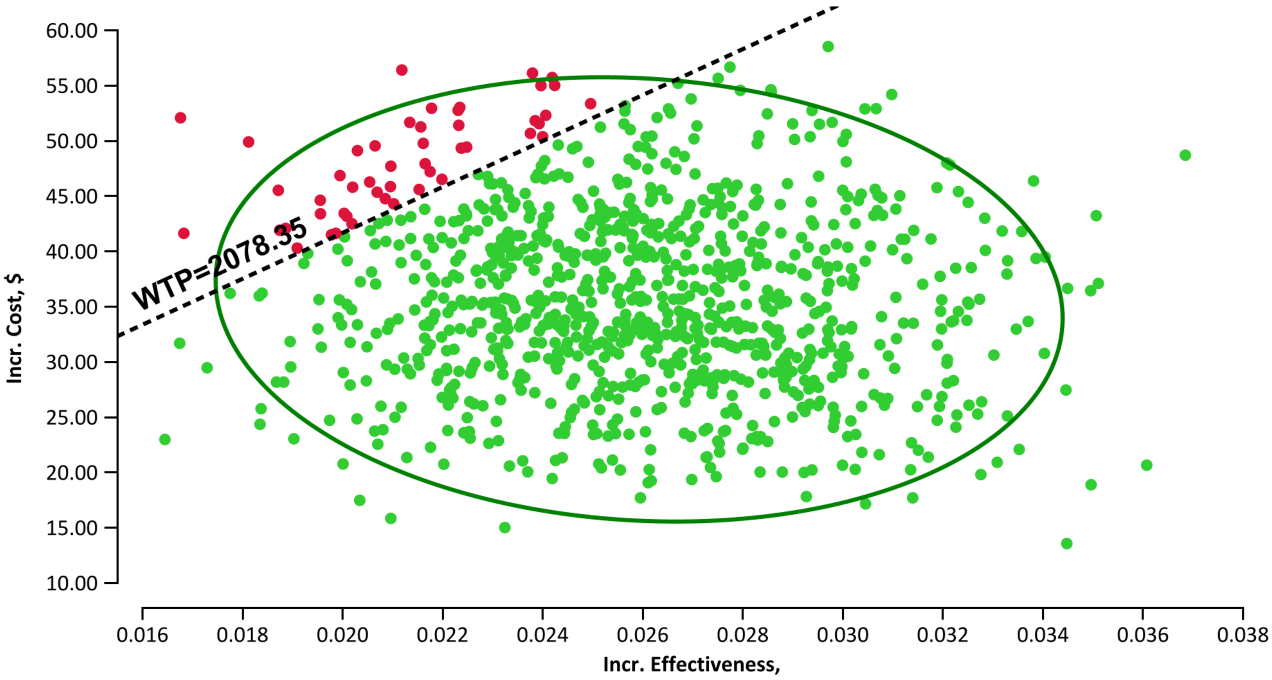

** Each point (green dots) represents a simulation from the probabilistic sensitivity analysis. WTP, Willingness-To-Pay representing cost-utility threshold, Incre, Incremental*

Table S4. Scatter plot report: Incremental cost-utility analysis of FCM versus FS

Component	Quadrant	Incremental Cost	Incremental Effectiveness	ICUR	Frequency	Proportion
C1	IV	IC<0	IE>0	Superior	0	0
C2	I	IC>0	IE>0	ICER<2078.35	956	0.956
C3	III	IC<0	IE<0	ICER>2078.35	0	0
C4	I	IC>0	IE>0	ICER>2078.35	44	0.044
C5	III	IC<0	IE<0	ICER<2078.35	0	0
C6	II	IC>0	IE<0	Inferior	0	0
Indifference	origin	IC=0	IE=0	0/0	0	0

IC, Incremental Cost, IE = Incremental Effectiveness, ICUR, Incremental Cost-Utility Ratio.

Quadrants refer to the standard cost-effectiveness plane: I = More effective and more costly, II = Less effective and more costly.

III = Less effective and less costly, IV = More effective and less costly.

**Probabilistic sensitivity analysis using 1.000 iterations*

References

1. MapChart. Nigeria Map [Internet]. MapChart. [cited 2025 Jun 25]. Available from: https://www.mapchart.net/africa.html

2. World Bank Group. World Bank Open Data [Internet]. World Bank national accounts data, and OECD National Accounts data files. 2023 [cited 2025 May 17]. Available from: https://data.worldbank.org/indicator/NY.GDP.DEFL.KD.ZG
3. The Center for Clinical Trials R and IS. Comprehensive Approach to the Prevention and Management of Maternal Anaemia in Nigeria (CAPREMAN) [Internet]. CCTRIS. 2025 [cited 2025 Jun 25]. Available from: cctris.org/research-projects/
4. Indiamart. Ferric carboxymaltose Products: Different estimates [Internet]. IndiaMART. [cited 2025 Jun 22]. Available from: https://www.indiamart.com/proddetail/ferric-carboxymaltose-injection-500mg-10ml-22762401162.html

5. Auerbach M, Gafter-Gvili A, Macdougall IC. Intravenous iron: a framework for changing the management of iron deficiency. Lancet Haematol. 2020;(4):e342–50.

6. World Health Organization. WHO-CHOICE estimates of cost for inpatient and outpatient health service delivery [Internet]. 2021 [cited 2025 Jun 29]. Available from: https://www.google.com/url?sa=t&source=web&rct=j&opi=89978449&url=https://www.who.int/publications/m/item/who-choice-estimates-of-cost-for-inpatient-and-outpatient-health-service-delivery&ved=2ahUKEwiiq_rbipaOAxXYWUEAHUYUAasQFnoECBsQAQ&usg=AOvVaw0TXdKx-7iL3cSrf4TY0GgB

7. Akinajo OR, Banke-Thomas A, Annerstedt KS, Beňová L, Adelabu YA, Sam-Agudu NA, et al. Intravenous iron for anaemia in pregnancy: A quantitative study of acceptability and feasibility of its integration into routine antenatal care practice in Nigeria. PLoS One. 2026; 21(1): e0328239.

8. Sharan AD, Schroeder GD, West ME, Vaccaro AR. Understanding Time-driven Activity-based Costing. Clin Spine Surg. 2016;29(2):62–5.

9. Drummond MF, Sculpher MJ, Claxton K, Stoddart GL, Torrance GW. Methods for the Economic Evaluation of Health Care Programmes. 4th ed. Oxford: Oxford University Press. 2015. Available from: pure.york.ac.uk/portal/en/publications/methods-for-the-economic-evaluation-of-health-careprogrammes (8f69bce
10. Ashaolu AT, Adeoye IA. Incidence, risk factors and outcomes of neonatal near miss in Lagos, Nigeria: a prospective case-control study. BMC Pediatr. 2025 Dec 1;25(1):869.

11. Setiawan E, Cassidy-Seyoum SA, Thriemer K, Carvalho N, Devine A. A Systematic Review of Methods for Estimating Productivity Losses due to Illness or Caregiving in Low- and Middle-Income Countries. Vol. 42, Pharmacoeconomics. 2024;42(8):865–77.

12. Global Burden of Disease Collaborative Network. Global Burden of Disease Study 2019 (GBD 2019) Disability Weights. Seattle, United States of America: Institute for Health Metrics and Evaluation (IHME), 2020. [Internet]. 2020 [cited 2024 Nov 21]. Available from: https://doi.org/10.6069/1W19-VX76

13. Benson CS, Shah A, Frise MC, Frise CJ. Iron deficiency anaemia in pregnancy: A contemporary review. Obstet Med. 2021;14(2):67–76.

14. Ba DM, Ssentongo P, Kjerulff KH, Na M, Liu G, Gao X, et al. Adherence to Iron Supplementation in 22 Sub-Saharan African Countries and Associated Factors among Pregnant Women: A Large Population-Based Study. Curr Dev Nutr. 2019;(12):1–8.
15. Afolabi BB, Babah OA, Adeyemo TA, Balogun M, Banke-Thomas A, Abioye AI, et al. Intravenous versus oral iron for anaemia among pregnant women in Nigeria (IVON): an open-label, randomised controlled trial. Lancet Glob Health. 2024;12(10):e1649–59.

16. Pasricha SR, Mwangi MN, Moya E, Ataide R, Mzembe G, Harding R, et al. Ferric carboxymaltose versus standard-of-care oral iron to treat second-trimester anaemia in Malawian pregnant women: a randomised controlled trial. The Lancet. 2023;401(10388):1595–609.

17. Breymann C, Milman N, Mezzacasa A, Bernard R, Dudenhausen J. Ferric carboxymaltose vs. oral iron in the treatment of pregnant women with iron deficiency anemia: An international, open-label, randomized controlled trial (FER-ASAP). J Perinat Med. 2017;(4):443–53.

18. Institute for Health Metrics and Evaluation. Maternal disorders [Internet]. 2020 [cited 2025 Jan 5]. Available from: www.healthdata.org/sites/default/files/files/policy_report/2022/GBD_2020_methods_maternal_disorders.pdf

19. Eddy DM, Hollingworth W, Caro JJ, Tsevat J, McDonald KM, Wong JB. Model transparency and validation: A report of the ISPOR-SMDM modeling good research practices task force-7. Medical Decision Making. 2012;32(5):733–43.
