# Supplementary material for: Intravenous ferric carboxymaltose versus oral ferrous sulphate for iron deficiency anaemia in pregnancy in Nigeria: a cost-utility analysis
Source: Lancet Obstet Gynaecol Womens Health. 2026 Jun;2(6):e524–34. doi: 10.1016/S3050-5038(26)00049-X (PMC13288457; doi:10.1016/S3050-5038(26)00049-X)
Supplement: Equitable Partnership Declaration [file mmc2.pdf]

THE LANCET
Obstetrics, Gynaecology, & Women's Health

Supplementary appendix 2

This Equitable Partnership Declaration (EPD) was submitted by the authors, and we reproduce it as supplied. It has not been peer reviewed. *The Lancet*'s editorial processes have not been applied to the EPD.

Supplement to: Akinajo OR, Annerstedt KS, Santos MT, Afolabi BB, Banke-Thomas A. Intravenous ferric carboxymaltose versus oral ferrous sulphate for iron deficiency anaemia in pregnancy in Nigeria: a cost-utility analysis. *Lancet Obstet Gynaecol Womens Health* 2026; published online April 28. https://doi.org/10.1016/S3050-5038(26)00049-X.

Equitable Partnership Declaration

If any questions do not apply to your study, please indicate "N/A" for "not applicable.
For more information on how to complete this form see the Information for Authors document.

Researcher considerations

1. Please detail the involvement that researchers who are based in the country or countries of study had during a) study design; b) clinical study processes, such as processing blood samples, prescribing medication, or patient recruitment; c) data interpretation; and d) manuscript preparation, commenting on all aspects. If they were not involved in any of these aspects, please explain why.

   This should include a thorough description of their leadership roles in the study. Are local researchers named in the author list or the acknowledgements, or are they not mentioned at all (and, if not, why)? Please also describe the involvement of early career researchers based in the location of the study. Some of this information might be repeated from the Contributors section in the manuscript. Note: we adhere to [ICMJE authorship criteria](#) for naming authors on a paper.

**a) Study design:**<br><br>ORA, AB-T, KSA, and BBA were involved in the conceptualisation of the research ideas, study design and in reviewing and approving the research title and proposal
**b) Clinical study processes:**<br><br>Not applicable
**c) Data interpretation:**<br><br>ORA, AB-T, KSA, and MTS interpreted data analysis. BBA was the principal investigator of the IVON trial from which this study was extracted and contributed to data interpretation.
**d) Manuscript preparation:**<br><br>ORA prepared the research manuscript under the supervision of AB-T (lead supervisor), KSA, and BBA, with contributions from MTS. All authors critically revised and endorsed the last version of the manuscript for publication.

2. How was funding used to remunerate and enhance the skills of researchers in the countries of study? And how was funding used to improve research infrastructure at the study sites?

   Potentially effective investments into long-term skills and opportunities within local institutions could include training or mentorship in analytical techniques and manuscript writing, opportunities to lead all or specific aspects of the study, financial remuneration rather than requiring volunteers, and other professional development and educational opportunities.

   Improvements to research infrastructure could include funding extended trial designs (eg, platform trials), establishment of long-term contracts for research staff, building research facilities, and setting up local control of funding allocation.

**Skills:**<br><br>Funding enabled local researchers to participate through appropriate remuneration, allowing them to dedicate time to the study without relying on voluntary contributions. For example, research assistants were recruited and received structured training in data collection using multiple methods, including time-based costing approaches. Their sustained engagement through funded roles contributed to skill development and strengthened research capacity within the team.<br><br>As an early-career researcher in economic evaluation, I also benefited from the funding through capacity development. I received training in economic evaluation methods, including analytical approaches and data analysis techniques. This included hands-on training in TreeAge software, enabling me to build decision tree models and conduct analyses using various cost and health-outcome parameters, including sensitivity analyses.
**Research infrastructure:**<br><br>Funding supported the study's implementation within existing institutional systems by enabling the recruitment and sustained engagement of research staff, thereby improving continuity and coordination of study activities. It facilitated data collection and management, including the use of structured data collection tools and costing templates, which strengthened the organisation and quality of study data.<br><br>In addition, the funding supported the establishment and use of standardised data management and documentation systems, including structured databases and data tracking processes, which improved data quality, accessibility, and consistency across the study. It also strengthened operational workflows for study coordination, monitoring, and reporting, thereby enhancing the overall efficiency and sustainability of research activities at the study sites.

3. How did you safeguard the researchers who implemented the study?

   Please describe how you guaranteed safe working conditions for study staff, including provision of appropriate personal protective equipment, protection from violence, and prevention of overworking.

Measures were put in place to ensure safe and supportive working conditions for all research staff. Work responsibilities were clearly defined and distributed among team members to prevent excessive workload, and timelines were structured to allow adequate time for task completion without undue pressure. Research staff were engaged on a remunerated basis, which supported accountability while avoiding reliance on voluntary or unpaid work that could contribute to overworking.<br><br>Regular team check-ins and supervision were conducted to monitor workload, provide support, and address any challenges encountered during the study. Data collection activities were scheduled in coordination with routine clinical workflows to minimise disruption and reduce stress on staff. Overall, these measures helped promote a balanced workload, safeguard staff wellbeing, and maintain a safe working environment throughout the study.

<u>Benefits to the communities and regions of study</u>

4. How does the study address the research and policy priorities of its location?

How were the local priorities determined and then used to inform the research question? Who decided which priorities to take forward? Which elements of the study address those priorities?

This study addresses a key local priority of improving the management of moderate to severe iron-deficiency anaemia (IDA) in pregnancy by evaluating the cost-effectiveness of ferric carboxymaltose (FCM) compared to ferrous sulphate (FS). Given the high burden of IDA and resource constraints in the study setting, there is a clear need for evidence to guide efficient allocation of limited healthcare resources.

The research question was informed by existing clinical practice challenges, including suboptimal treatment outcomes with oral iron and the higher upfront cost of intravenous iron, as well as national and global recommendations on anaemia management. These priorities were identified through engagement with local clinicians, researchers, and existing evidence, and were jointly agreed upon by the study team, with strong input from local investigators.

By demonstrating the cost-effectiveness of FCM relative to FS, the study provides context-specific evidence to support policy decisions on integrating FCM into routine antenatal care. In addition, the findings highlight the importance of reducing the cost of FCM to improve affordability and promote equitable access to effective treatment, thereby informing pricing and procurement strategies within the health system.

5. How will research products be shared in the community of study?

For instance, will you be providing written or oral layperson summaries for non-academic information sharing? Will study data be made available to institutions in the region(s) of study? The Lancet Global Health *encourages authors to translate the summary (abstract) into relevant languages after paper editing; do you intend to translate your summary?*

Findings from this study will be shared through both academic and non-academic channels to ensure accessibility to a broad audience. In addition to publication in a peer-reviewed journal, key results will be communicated to local stakeholders, including clinicians, hospital administrators, and policymakers, through presentations and stakeholder engagement meetings.

To support non-academic dissemination, simplified summaries of the findings will be prepared to communicate key messages in an accessible format for broader audiences. Where feasible, these summaries may be shared through institutional platforms and relevant professional networks.

Study findings will also be made available to relevant institutions within the study setting to support local decision-making and policy development. In line with journal recommendations, we are open to translating the study summary into relevant local languages to enhance accessibility and community engagement.

6. How were individuals, communities, and environments protected from harm?

a) *How did you ensure that sensitive patient data were handled safely and respectfully? Was there any potential for stigma or discrimination against participants arising from any of the procedures or outcomes of the study?*

Measures were implemented to ensure that participants and study environments were protected from harm throughout the study. Data collection involving participants was conducted using structured tools designed to minimise burden and avoid disruption to routine care. Informed consent was obtained from all participants prior to data collection, and participation was entirely voluntary.

Sensitive patient data were handled in accordance with established ethical and data protection standards. No personally identifiable information was collected, and all data were anonymised prior to analysis. Data were securely stored, with access restricted to authorised members of the research team.

The study posed minimal risk of stigma or discrimination, and did not involve sensitive personal or social characteristics. All findings were reported in aggregate form, ensuring that no individual participant could be identified.

b) *Might any of the tests be experienced as invasive or culturally insensitive?*

Not applicable

c) *How did you determine that work was sensitive to traditions, restrictions, and considerations of all cultural and religious groups in the study population?*

The study was designed and implemented with consideration for local cultural and religious practices. Data collection was conducted in routine clinical settings by trained local research staff who were familiar with the study population's cultural context and language. This helped ensure that interactions with participants were respectful and appropriate.

Study procedures, including consent and data collection, were carried out in a manner that respected participants' preferences, privacy, and comfort. No procedures in the study conflicted with cultural or religious practices, as data collection was limited to routine care settings and focused on non-invasive information.

Ongoing engagement with local clinicians and investigators further ensured that the study remained sensitive to cultural and contextual considerations throughout its implementation.

d) *Were biowaste and radioactive waste disposed of in accordance with local laws?*

Not applicable

e) *Were any structures built that would have impacted members of the community or the environment (such as handwashing facilities in a public space)? If so, how did you ensure that you had appropriate community buy-in?*

Not applicable

f) *How might the study have impacted existing health-care resources (such as staff workloads, use of equipment that is typically employed elsewhere, or reallocation of public funds)?*

Not applicable

7. Confirm that local ethics review was sought, and please provide the approval number. If not sought, please explain why.

This study was conducted within the context of the main clinic trial (the IVON trial). Before the conduct of that trial, ethical approval was obtained from National Health Research Ethics Committee (NHREC/01/01/2007- 04/02/2021), Lagos University Teaching Hospital Health Research Ethics Committee (ADM/DCST/HREC/ APP/3971), Kano State Ministry of Health (MOH/ Off/797/T.1/2102), and Aminu Kano Teaching Hospital (NHREC/28/01/2020/AKTH/EC/2955).<br><br>Permission to conduct research in the secondary and primary health facilities in Lagos State was obtained from the Lagos State Health Service Commission (LSHSC/2222/VOLIII) and the Lagos State Primary Health Care Board (LS/PHCB/MS/1128/VOL.VII/100), respectively.

Secondary analyses

8. Have the data analysed in your study been extracted from another source, such as a national survey, rather than being directly collected by the authors of this paper?

Yes

If the authors of this paper were not involved in data collection, how were the findings interpreted with sufficient contextual knowledge?
The Lancet Global Health *believe contextual understanding is crucial for informed data analysis and interpretation.*

In addition to primary data collection on costs from study participants using multiple approaches, data were also sourced from secondary literature, including the main trial. These secondary data were identified and selected using a graded approach to ensure quality, based on study design, relevance, validity, and consistency of reported outcomes.<br><br>Contextual understanding was ensured through the involvement of study team members who were familiar with the study setting, population, and clinical practices, including those involved in primary data collection. All authors further contributed to the interpretation of findings, ensuring that assumptions, parameter estimates, and results were appropriate and relevant to the local context and health system.

---

9. Finally, please provide the title (eg, Dr/Prof, Mr/Mrs/Ms/Mx), name, and email address of an author who can be contacted about this statement.

Name: Dr Opeyemi Rebecca Akinajo
Email: oakinajo.cctris@unilag.edu.ng
